# Pressure driven spin transition in siderite and magnesiosiderite single crystals

**DOI:** 10.1038/s41598-017-16733-3

**Published:** 2017-11-28

**Authors:** Christopher Weis, Christian Sternemann, Valerio Cerantola, Christoph J. Sahle, Georg Spiekermann, Manuel Harder, Yury Forov, Alexander Kononov, Robin Sakrowski, Hasan Yavaş, Metin Tolan, Max Wilke

**Affiliations:** 1 Fakultät Physik/DELTA, Technische Universität Dortmund, Dortmund, 44227 Germany; 2 European Synchrotron Radiation Facility, 71 Avenue des Martyrs, Grenoble, 38000 France; 3 Institute of Earth and Environmental Science, Universität Potsdam, Potsdam, 14476 Germany; 4 Deutsches Elektronen-Synchrotron DESY, Hamburg, 22607 Germany

## Abstract

Iron-bearing carbonates are candidate phases for carbon storage in the deep Earth and may play an important role for the Earth’s carbon cycle. To elucidate the properties of carbonates at conditions of the deep Earth, we investigated the pressure driven magnetic high spin to low spin transition of synthetic siderite FeCO_3_ and magnesiosiderite (Mg_0.74_Fe_0.26_)CO_3_ single crystals for pressures up to 57 GPa using diamond anvil cells and x-ray Raman scattering spectroscopy to directly probe the iron 3d electron configuration. An extremely sharp transition for siderite single crystal occurs at a notably low pressure of 40.4 ± 0.1 GPa with a transition width of 0.7 GPa when using the very soft pressure medium helium. In contrast, we observe a broadening of the transition width to 4.4 GPa for siderite with a surprising additional shift of the transition pressure to 44.3 ± 0.4 GPa when argon is used as pressure medium. The difference is assigned to larger pressure gradients in case of argon. For magnesiosiderite loaded with argon, the transition occurs at 44.8 ± 0.8 GPa showing similar width as siderite. Hence, no compositional effect on the spin transition pressure is observed. The spectra measured within the spin crossover regime indicate coexistence of regions of pure high- and low-spin configuration within the single crystal.

## Introduction

The potential subduction of carbon into the deep Earth involves still many unsolved questions that are essential for understanding the Earth’s carbon cycle^1^. Owing to the low solubility of carbon in the Earth’s main mantle silicate phases^2,3^, carbon is stored in accessory phases such as carbonates^4^, methane^5^, carbides^6^, and diamonds^5^ depending mainly on temperature, pressure and oxygen fugacity. Carbonate minerals are abundant at the Earth’s surface and their crystal chemistry and mineralogy at ambient conditions was investigated in detail over the past decades (see e.g.^7^). Due to subduction of oceanic crust at convergent plate-boundaries, carbonates may be transported into the interior of the Earth^8^ and indeed their occurrence in the upper and lower mantle was evidenced by inclusions in diamonds of super-deep origin^4,9–11^. Various carbonates, which are present in the Earth’s mantle are stable over a broad temperature and pressure range without decomposing. For example, MgCO_3_ was found to be stable up to 115 GPa and 2100 K^12^. Consequently, the properties of the carbon host minerals FeCO_3_ (siderite), MgCO_3_ (magnesite) and CaCO_3_ (calcite) at high pressure and high temperature, as well as the corresponding solid solutions, were widely studied^12–23^. In solid solutions formed by magnesite and siderite, pressure and temperature induced changes of structure and electronic properties are largely determined by iron^16,20,24^.

In contrast to other major Earth elements, iron has partially filled 3d electronic orbitals that define the magnetic state of the iron-bearing carbonates. Fe^2+^ undergoes a transition from the high-spin (HS) to low-spin (LS) state at pressure conditions of the Earth’s lower mantle^25,26^. This transition is associated with a volume collapse and thus significantly influences the macroscopic properties of iron-bearing minerals such as sound velocity, conductivity and compressibility, as well as the chemical behavior of iron itself^27^. In order to better understand the role of iron-bearing carbonates in processes of the deep Earth, precise knowledge about the effect of pressure and temperature on the electronic state of iron in these phases is needed.

Mattila *et al*.^28^ found the spin transition of natural siderite (Fe_0.96_Mn_0.04_)CO_3_ to occur at roughly 50 GPa by means of K*β* x-ray emission spectroscopy (XES). Density-functional theory (DFT) calculations indicated that the magnetic transition appears between 15 to 28 GPa^29^, which is considerably lower in comparison to the results of Mattila. Later, Lavina *et al*.^30^ observed the corresponding spin change induced isostructural volume collapse of 10 % in natural siderite (near end-member) by x-ray diffraction (XRD) in a narrow pressure range between 44–45 GPa, associated with a shrinkage of the octahedral Fe-O bond distance by 4 %. Lobanov *et al*.^31^ found the spin transition to take place in between 44 and 45 GPa by means of visible and near infrared absorption spectroscopy of (Fe_0.95_Mn_0.05_)CO_3_. However, a coherent picture of this transition was not achieved in the following years. Measurements performed by Nagai *et al*.^32^ by means of XRD and by Farfan *et al*.^33^ with a combination of optical Raman spectroscopy and XRD on natural siderite (Fe_0.73_Mg_0.22_Mn_0.05_)CO_3_ and (Fe_0.76_Mn_0.15_Mg_0.09_Ca_0.01_)CO_3_, respectively, showed the spin transition to occur at pressures between 46 and 50 GPa, while Lin *et al*.^16^ determined a value of 45 GPa in (Fe_0.65_Mg_0.35_)CO_3_ exploiting the same methods. Lavina *et al*.^34^ revealed the spin transition of (Mg_0.87_Fe_0.12_Ca_0.01_)CO_3_ to take place between 49 and 52 GPa using XRD. Later, Liu *et al*.^17^ reported a shift of the spin transition to higher pressures and a broadening of the transition range as a function of temperature in magnesiosiderite (Fe_0.65_Mg_0.33_Mn_0.02_)CO_3_.

In order to exclude effects of chemical composition on the course of the transition in natural samples, first studies on synthetic siderite were conducted by Spivak *et al*.^35^ using optical Raman spectroscopy and by Cerantola *et al*.^18^ applying a combination of Mössbauer, optical Raman and x-ray absorption (XAS) experiments. Both found that the spin transition proceeds gradually over a broad pressure range of 40–46 GPa. They suggested a coexistence regime of HS and LS iron until complete transformation. These results have challenged earlier XRD data, which observed a very narrow pressure range, as indicated by volume collapse. Most recently Müller *et al*.^22^ showed that pressure gradients within the sample volume may be the major reason for a broad transition range. They observed the transition between 43.3 and 45.5 GPa, together with the coexistence of HS and LS regimes due to pressure gradients.

The inconclusive reports on transition pressure and transition range, both for the end-member siderite and the magnesiosiderite solid solution, led to controversial discussions with regard also to the compositional effect on the spin transition^17,34–36^. While Spivak *et al*.^35^ and Lavina *et al*.^34^ observed that the amount of iron in magnesiosiderite samples significantly influences the course of the spin transition, Liu *et al*.^17^ and Hsu *et al*.^36^ found no compositional effect on the spin transition pressure. Lately, it was reported by Müller *et al*.^37^ that at ambient temperature the spin transition pressure is hardly affected by the composition while at higher temperature the transition range significantly broadens for (Fe_0.24_Mg_0.76_)CO_3_ compared to FeCO_3_.

In contrast, the spin transition in the magnesiowustite solid solution shows considerable dependence on the iron-content at ambient temperature^17^. Therefore, a detailed characterization of the spin state of siderite and its solid solution with magnesite is demanded using complementary approaches that especially probe directly the electronic state of the system such as e.g. XAS at the iron K-edge^38^ and iron K*β*_1,3_ or valence-to-core XES^39^.

In this work we exploit x-ray Raman scattering (XRS) spectroscopy to study the iron M_2,3_-edges^40^ and L_2,3_-edges^41^ of siderite and magnesiosiderite *in situ* at high pressure in a diamond anvil cell (DAC) in order to probe the electronic structure of iron. XAS measurements at the iron L_2,3_-edges (2p→3d) for light- or temperature induced spin transitions have shown that the L_2,3_-edge is highly sensitive to the spin state^42,43^, but high pressure experiments using DAC prevent the use of soft x-ray absorption for the *in situ* study of the L_2,3_- and/or M_2,3_-edges of iron in the carbonates due to the strong absorption of soft x-rays by the diamond anvils. The advantage of XRS^44^, a non-resonant inelastic x-ray scattering technique, is that it allows probing low energy absorption edges by hard x-rays^45–48^. The method is sensitive to local coordination^49^ and oxidation state^50^. Lately, Nyrow *et al*.^40^ demonstrated the sensitivity of XRS with respect to the spin state by measuring the iron M_2,3_-edge of FeS powder at high pressure. In this study we investigate the pressure-induced HS to LS transition of synthetic siderite and magnesiosiderite single crystals at the iron M_2,3_-edge and iron L_2,3_-edge in the pressure range up to 57 GPa using argon and helium as pressure medium at room temperature by means of XRS. We were able to track the spin state change at both absorption edges and clearly identify transition pressure, transition range, and effect of composition.

## Results and Discussion

**Figure 1:**
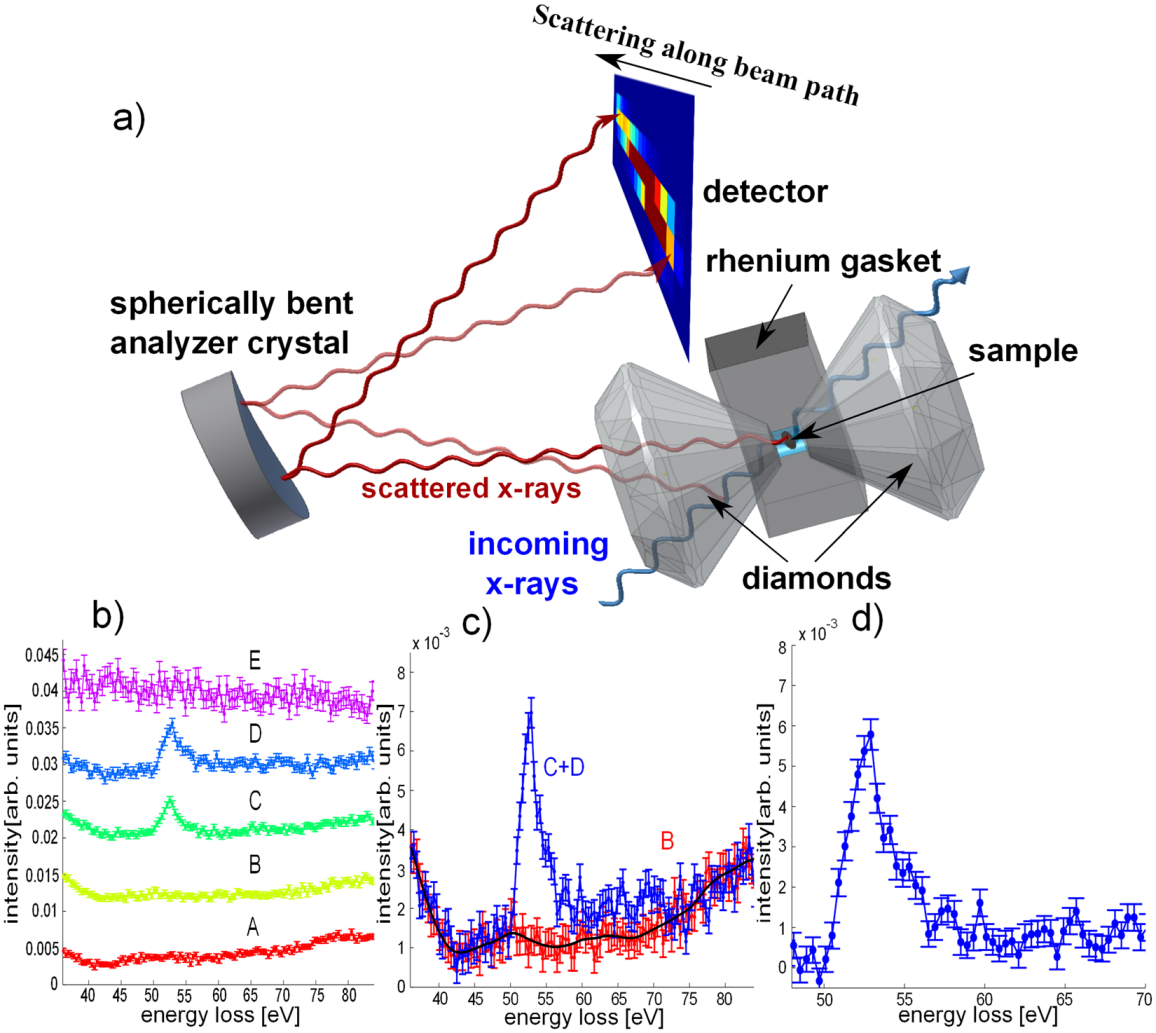
Imaging properties of XRS and data extraction. (**a**) Schematic sketch of the scattering geometry for the high pressure XRS measurements. The inelastically scattered photons from each point of the x-ray beam path through sample and diamond are analyzed and focused onto different positions on the 2D detector. (**b)** XRS spectra for energy losses at the iron M_2,3_-edge obtained from a single pixel analysis along the beam path. Spectra A and B show the signal of the diamond before the sample, curves C and D contain contributions from diamond and sample and spectrum E shows the signal of the diamond behind the sample, which is partly masked by the gasket. (**c)** Summed signals for selected pixels which contain the background signal only (red, B) and spectra from pixel that contain sample and background signal (blue, summation of pixel C & D). The black solid line indicates the smoothed background signal. (**d)** Extracted XRS M_2,3_-edge after subtraction of the smoothed background.

**Figure 2:**
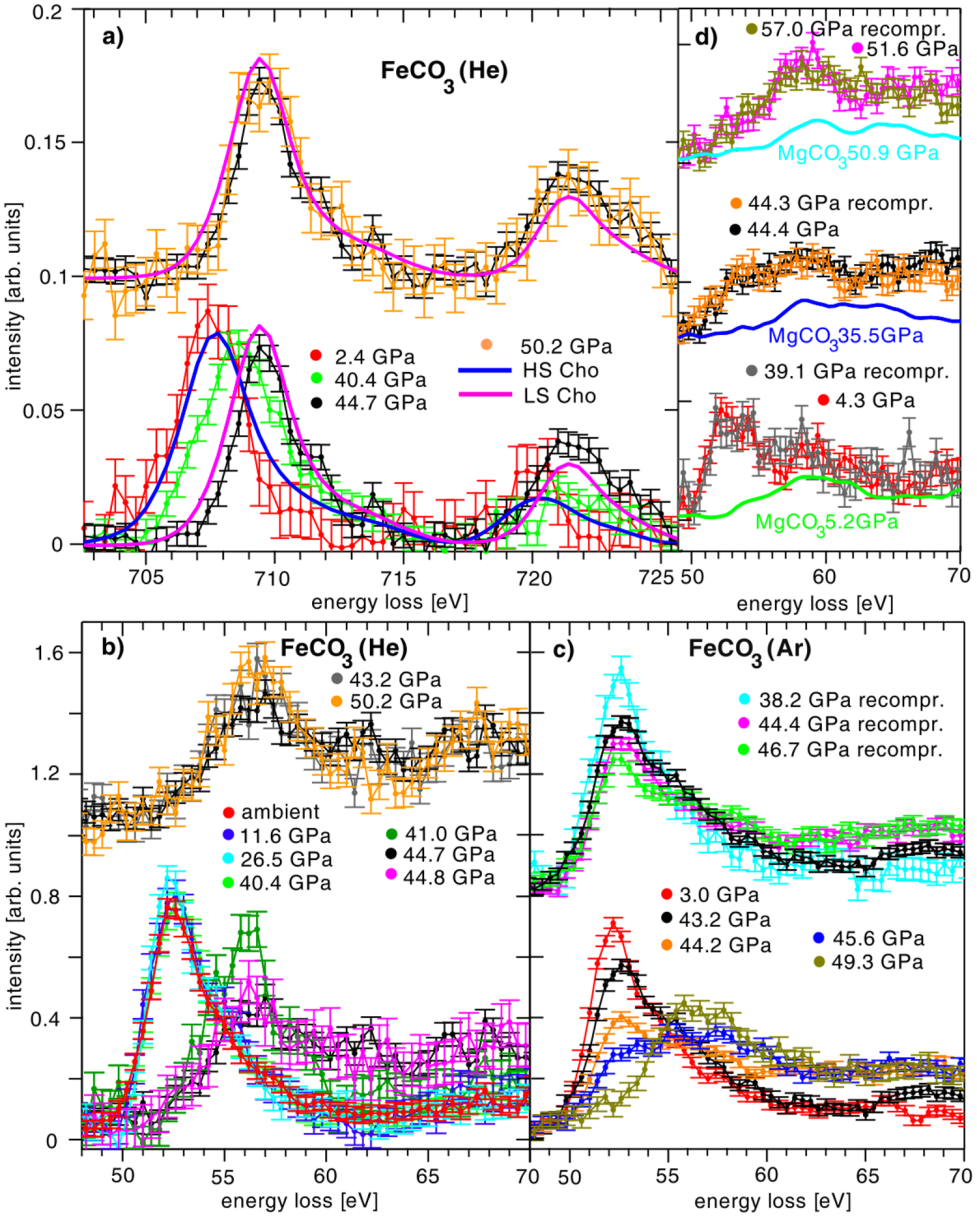
(**a)** Iron L_2,3_-edge XRS spectra measured at low momentum transfer (3.2 ± 0.9 Å^−1^) with helium as pressure medium. The data for 2.4 GPa and 40.4 GPa have been published in^41^. References for Fe^2+^ L_2,3_-edge spectra of a [Fe(tren(py))_3_]^2+^ measured by means of soft x-ray absorption for the high and low spin state taken from^42^ are shown as solid lines. *In situ* XRS spectra of M_2,3_-edges of siderite single crystal as a function of pressure using helium (**b)** and argon (**c)** as pressure medium (12.7 ± 0.2 Å^−1^). Siderite powder was measured at ambient pressures enclosed in a DAC as a reference without pressure medium. (**d**) Iron XRS M_2,3_-edges of magnesiosiderite (Mg_0.74_ Fe_0.26_)CO_3_ single crystal (dots with errorbars) and magnesium XRS L_2,3_-edges of magnesite powder (solid lines).

A schematic sketch of the experimental setup and the scattering geometry of XRS using DACs in backscattering mode is shown in Fig. 1. The incoming photons penetrate the first diamond, the sample, which is enclosed in a rhenium gasket, and the second diamond. From each point along the beam path photons are inelastically scattered into the direction of the spherically bent analyzer crystal and are then analyzed at different positions of a 2D detector. This point-to-point focusing generates an image of the sample and sample environment, projected onto the detector with an intensity contrast according to the corresponding XRS cross section (see for details^51,52^). We exploit the imaging properties of XRS (see^51,53,54,55^) to separate the sample signal from the complex background, which consists of contributions from either diamonds and/or the gasket, which is exemplified in Fig. 1(b–d) and is discussed in the methods section. The background corrected XRS spectra were normalized to the integrated intensity in the energy loss range from 49.7–65 eV and 702.2–726.6 eV for M_2,3_ and L_2,3_-edge, respectively. Figure 2 shows the iron M_2,3_ and L_2,3_-edges of siderite, and the M_2,3_-edges of magnesiosiderite single crystal for various pressures. We will use the spectra to determine the position and width of the transition, and compare the results with argon and helium as pressure medium. The compositional effect is then investigated for magnesiosiderite (Mg_0.74_ Fe_0.26_)CO_3_. Finally, a component fit of the XRS spectra employing high- and low-spin references is used to investigate the electronic state in the spin crossover regime.

### Siderite

First, we will discuss the XRS measurements using helium as pressure medium. The changes of the L_2,3_-edge with pressure are presented in Fig. 2(a). The L_3_-edge peak position shifts from 707.4 eV to 709.4 eV and the L_2_-edge from 720.0 eV to 721.4 eV. This shift is accompanied by a change in intensity between both edges. The pressure-induced changes of the L_2,3_-edges’ shape in the HS and LS states measured at 2.4 GPa and at 44.7 GPa, respectively are in line with soft-XAS spectra measured before and 150 ps after photoexcitation of the spin transition in [Fe(tren(py))_3_]^2+^ polypyridyl complexes^42^. Owing to the differences in energy resolution between the XAS and XRS spectra, the XAS spectra were convoluted with a gaussian of 1.7 eV FWHM for comparison. We estimate the L_3_/L_2_ branching ratio to change from 3.2 ± 0.8 to 1.6 ± 0.1 across the spin transition for the HS and LS state, respectively. These values have been estimated by using a bin of ±5 eV around the L_3_ and L_2_ peak positions. The HS value is in accordance with values obtained by Sparrow *et al*.^56^ for iron in HS configuration. A significant reduction of the branching ratio is observed in accordance with Thole *et al*.^57^. The spectrum measured at 40.4 GPa can be assigned to a sample state in the transition region. These findings confirm that the changes observed at the L_2,3_-edge can be directly related to the spin state of iron in siderite and allow us to assign the iron M_2,3_-edge spectral changes to HS and LS spin states as they were directly measured before the L_2,3_-edges. Regarding the relevance of L-edge studies in solid state physics and chemistry in general, we would like to note that the possibility of measuring L-edges of samples contained in a DAC paves the way to future studies of magnetic and electronic structure of e.g. transition metal compounds at high pressure.

The M_2,3_-edge of siderite in the HS state at low pressure shows a strong asymmetric peak at 52.5 eV. Notably, an abrupt disappearance of the characteristic HS peak in the M_2,3_-edge spectra (Fig. 2(b)) and a newly formed maximum at 56.5 eV indicates a very sharp spin transition between 40 GPa and 41 GPa. The M_2,3_-edge onset shifts from 51.1 eV in the HS to 53.8 eV in the LS state. Within error bars, only two types of M_2,3_-edge spectra can be distinguished, indicative for either HS or LS state. Strikingly, at 40.4 GPa the shape of M_2,3_-edge spectrum evidences iron to be in the HS state whereas the subsequently measured L_2,3_-edge indicates contributions from both HS and LS iron. Owing to the pressure increase during these measurements (from 39.9 to 40.9 GPa), the L_2,3_-edge spectrum was studied at slightly higher pressures thus shows a state right within the spin crossover pressure range supporting the spin transition to occur almost instantaneously in the run with helium as pressure medium.

To establish the reason for the broad transition range reported in^18,35^ we also measured the iron M_2,3_-edge at less hydrostatic conditions using argon as pressure medium to reveal details about the width of the spin transition. Here, we observed a gradual decrease of the HS peak at 52.5 eV with rising pressure in the pressure range of the spin crossover above 40 GPa and an increase of the LS maximum at 56.5 eV (Fig. 2c), bottom). Overall, the spectral shape varies over a broad pressure range in between 43.2–49.3 GPa. Top panel of Fig. 2(c) shows several spectra that were acquired in a second cycle after decompression to a pressure of 23 GPa. After re-pressurizing the sample, similar changes in the spectra are observed. The range of the spin crossover shifted to slightly higher pressures during the second compression cycle, due to the different hydrostatic conditions caused by the relaxation and contraction of the solid Argon.

### Magnesiosiderite

The iron M_2,3_-edges of magnesiosiderite (Mg_0.74_ Fe_0.26_)CO_3_ (argon was used as pressure medium to enable comparison with the corresponding siderite results) and magnesium L_2,3_-edges of magnesite MgCO_3_ powder are shown in Fig. 2(d). The iron M_2,3_-edge exhibits the same spectral changes (disappearance of the characteristic HS peak and shift of the spectral weight to higher energy loss values) as observed for the end-member siderite. Moreover, these changes take place at similar pressures as for siderite. The magnesite XRS spectra were collected in order to estimate the contribution of the Mg L_2,3_-edge underlying the iron M_2,3_-edge in magnesiosiderite. It can be seen that the shape of the Mg L_2,3_-edge hardly changes in the pressure range of the spin transition, indicating that the observed spectral changes of the magnesiosiderite spectra are caused solely by the change in the siderite spin state. In Fig. 2(d) the XRS spectra of magnesite were scaled such that the difference between the magnesiosiderite and the magnesite spectrum resembles the shape of the siderite M_2,3_-edge measured at 3.0 GPa. Our observations confirm the sensitivity of M_2,3_-edge XRS spectroscopy to spin states of compounds that have relevant phases in Earth’s mantle, even for cases with small amount of iron.

### Spin transition

**Figure 3:**
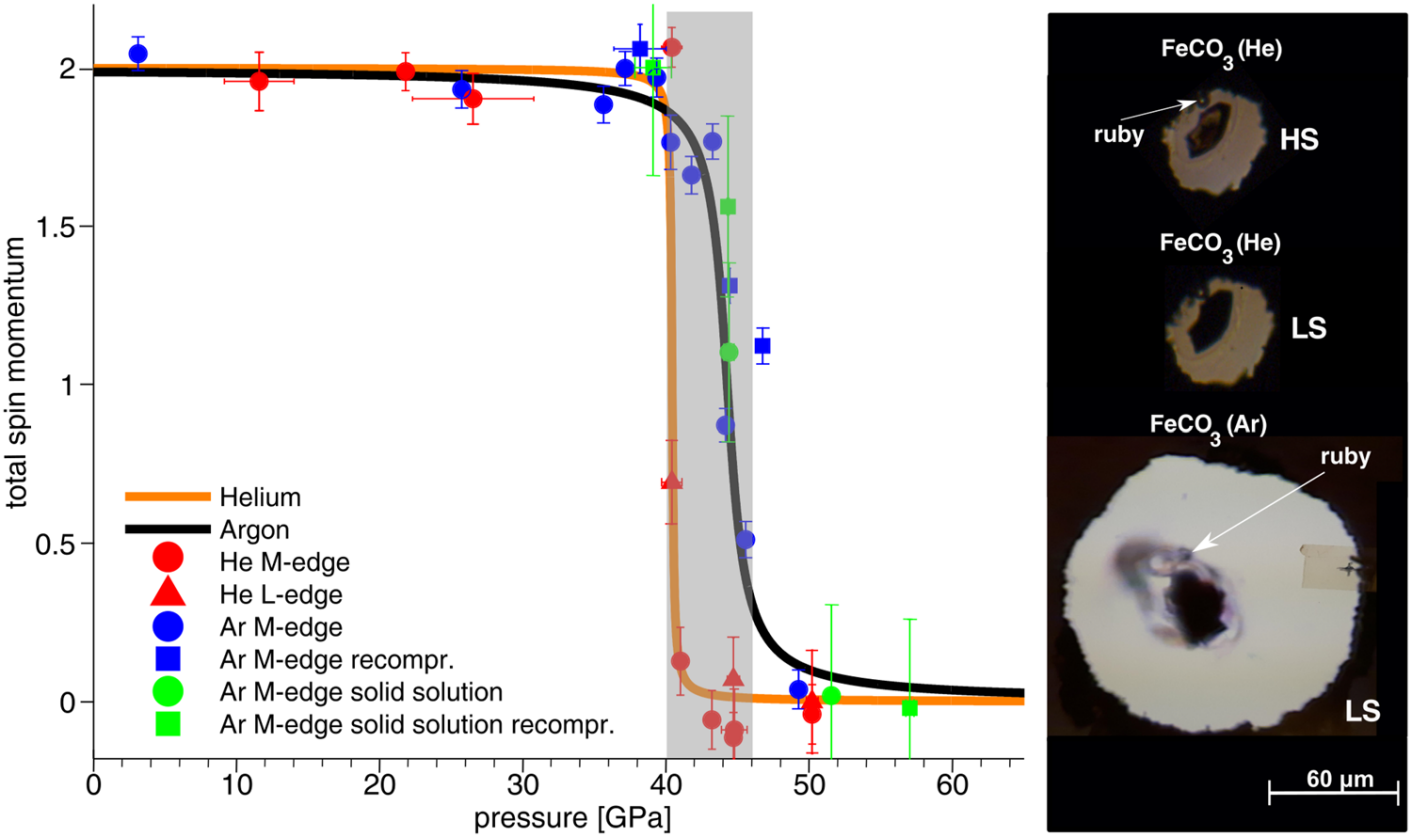
Left: Total spin momentum *S* as a function of pressure for all the spectra shown in Fig. 2. The grey shaded area (40–46 GPa) represents the coexistence regime of high- and low-spin iron revealed by Spivak *et al*.^35^. Right: Photographs of the siderite sample and ruby sphere position in the sample chamber for measurements with argon in the LS state and for measurements with helium in the LS and HS state.

In what follows, we present a scheme to analyze changes in the total spin momentum *S* of the compounds discussed above. As shown by Nyrow *et al*.^40^ the spin transition pressure and spin transition range can be characterized by the integral of the absolute value of *A*(*p*), which is given by the differences between spectra measured at certain pressure *p* and a HS reference measured at ambient conditions or low pressure. As our siderite reference, here we use the M_2,3_-edge and L_2,3_-edge measured at ambient conditions and 2.4 GPa, respectively. For the solid solution, we use the M_2,3_-edge spectrum measured at 4.3 GPa. As the Mg L_2,3_-edge hardly changes across the spin transition, its contribution almost cancels by calculating the difference spectra. The values of *A*(*p*) are finally normalized to 1 for the highest pressure in the LS state so that the total spin momentum *S* is obtained via *S* = 2 ⋅ (1−*A*(*p*)). The results of this analysis are depicted in Fig. 3.

In order to determine the transitions pressure and to analyze the width of the transition, *S* was fitted by an arctangent-function, separately for the experiments with different pressure media used in the measurements. The analysis reveals that the spin transition occurs at 44.3 ± 0.4 GPa for siderite single crystals with an overall transition range of about 4.4 GPa, in agreement with the results found in^18^ and^35^. With helium as pressure medium, which results in smaller pressure gradients^58^, the width of the transition is reduced to a range of about 0.7 GPa at 40.4 GPa. The difference in the width can well be assigned to smaller pressure gradients present for the helium medium. The additional shift of the transition by ca. 4 GPa for helium is quite surprising. There might be a systematic difference for the pressure measurement between the two runs, as illustrated by photographs of the sample chambers in Fig. 3 (right). For both experiments the ruby was placed next to the sample. In case of helium, a strong shrinkage of the sample chamber was observed, so that the ruby ended up next to the gasket. In this situation, the ruby may record a systematically lower pressure than present in the center of the gasket^59^. However, the observed shift exceeds the pressure variations reported in the literature^58,59^. Furthermore, some of the data points were recorded during an independent experiment revealing the helium loaded sample to be in the LS state at 44.8 GPa, which corroborates the view that this shift between argon and helium is real. For magnesiosiderite measured in argon, we estimate a transition pressure of 44.8 ± 0.8 GPa, which clearly shows that the composition hardly affects the phase transition at ambient temperature. Despite the reduced number of pressure points taken for the solid solution, we infer that the width of the transition is similar to that observed for siderite.

**Figure 4:**
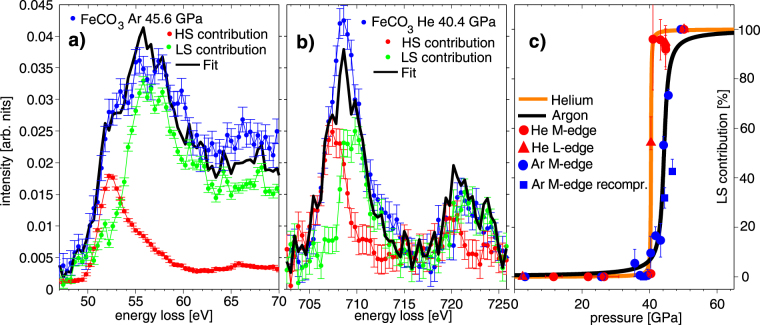
(**a**,**b)** Modeled siderite M_2,3_-edge (45.6 GPa) and L_2,3_-edge (40.4 GPa) measured within the spin crossover regime using a superposition of HS and LS reference spectra via component fit. (**c)** LS contribution to the component fits compared with the course of the spin transition as shown in Fig. 3.

Next, we investigate if spectra measured within the transition region can be modeled by a simple superposition of HS and LS spectra. In that case, rather than intermediate spin states, domains with sample in LS and HS states due to pressure gradient is assumed^13,36,60,61^. Hence, for siderite we fitted the XRS M_2,3_- and L_2,3_-edge spectra using a HS and LS reference. For the M_2,3_-edge, average XRS spectra measured at the highest and lowest pressures for argon-loaded and helium-loaded samples were used as HS and LS references, respectively. For the L_2,3_-edge, the spectrum measured at 50.2 GPa was used as the LS and 2.4 GPa as the HS reference. The results are shown exemplarily for a pressure of 45.6 GPa (M_2,3_-edge, argon series) and 40.4 GPa (L_2,3_-edge, helium series) in Fig. 4(a and b), respectively. The superposition of HS and LS references reproduces the shape of the spectra within the transition range for both edges. Consequently, a coexistence of LS and HS regions can be inferred and their change in weight is driven by pressure gradients in the sample. It has to be mentioned here that such analysis should preferentially be carried out for the data of helium runs due to the sharp transition range. However, evaluation of the spectra measured with argon yields consistent results providing additional evidence.

In Fig. 4(c), the contribution of the LS component to the total spectra is presented and confronted with the expected changes due to the course of the spin transition as discussed before. The error bars were calculated by variation of the fitting range of the component fit. A good overall agreement of the results obtained by the component fit with the direct analysis of the transition via *A*(*p*) is achieved and supports the interpretation in terms of coexistence of HS/LS regions in the pressure region of the transition, also in accordance with the results of Müller *et al*.^22^ and Cerantola *et al*.^18^ by means of optical Raman spectroscopy.

In this study we presented first *in situ* iron XRS M_2,3_- and L_2,3_-edge measurements of siderite FeCO_3_ and magnesiosiderite (Mg_0.74_ Fe_0.26_)CO_3_ single crystals exposed to high pressures up to 57 GPa at room temperature as a direct probe of the irons 3d electronic configuration. We observed strong pressure-induced spectral changes at both edges, which can be attributed to the HS to LS transition of iron, and were able to follow the spin transition with high sensitivity around the transition pressure. The detailed analysis by calculating the total spin momentum *S* on the basis of difference spectra indicates that an extremely sharp transition of Δ*p* = 0.7 GPa occurs at 40.4 ± 0.1 GPa for synthetic siderite single crystal loaded with helium. This transition pressure is significantly lower than observed so far in literature. Using argon as a pressure medium, we observe the transition at 44.3 ± 0.4 GPa and 44.8 ± 0.8 GPa for siderite and magnesiosiderite, respectively, with a broad spin crossover range of about Δ*p* = 4.4 GPa. The strong influence of the pressure medium is owed to the difference in hydrostatic behavior, inducing larger pressure gradients in the case of argon. From these experiments we infer that at room temperature the transition pressure is hardly affected by the composition of the solid solution. We were able to describe the XRS spectra of siderite measured within the spin crossover regime by a superposition of HS and LS reference spectra. This indicates that only parts of the single crystal crossed the spin transition and coexistence of HS and LS iron can be assumed without occurrence of iron in an intermediate 3d electronic configuration as discussed in^36,61^.

Enlightening the dynamics of the spin crossover in iron-bearing compounds is crucial for understanding the deep Earth’s or planetary dynamics. In particular, subducting slabs carrying iron-bearing carbonates have to be carried deeper than 1200 km (pressure that roughly correspond to 40 GPa) in order to completely reach the LS state. Our results concerning the pressure position, width of the transition as well as the influence of deviatoric stress imply that the spin crossover in the ductile lower mantle may be relatively sharp, if it is not washed out by temperature effects. The HS to LS transition will directly relate to the partitioning of iron between accessory carbonate and main mantle phases. Thus, owing to the lower transition pressure observed for iron-carbonates with respect to other main mantle minerals^62–64^, iron could partition preferentially into the carbonate structure when in LS state due to its smaller ionic radius with respect to Mg-atoms and iron in HS state in e.g. (Mg,Fe)O and bridgemanite^20^. Due to the absence of any composition-dependence on the spin crossover pressure this would also hold for carbonates closer in composition to calcite and magnesite end-members, those likely more representative for carbonates in subducting slabs. To conclude, carbonates at depths of mid-bottom lower mantle (above 40 GPa) could be strongly enriched in iron respect the surrounding minerals or carbonates at shallower pressures. However, these implications have to be confirmed by further experiments on temperature effects on the spin transition or experiments on iron partitioning among those phases at conditions present in the Earth’s mantle.

## Methods

### X-ray Raman scattering

XRS is a non-resonant inelastic x-ray scattering technique to perform bulk sensitive measurements of absorption edges in the soft x-ray regime using hard x-rays as probe, which finds unique applications in the field of high pressure research^45,47,65^. In such experiments, the incident photon with energy *ħω*_1_ (*ħω*_1_ is much larger than the electron binding energies of the excitation to be probed, i.e. in case of the iron M_2,3_-edge *ħω*_1_ is about 13000 eV while the binding energy is 52.5 eV) and wave vector **k**_**1**_ is inelastically scattered by excitation of a core electron, turning into a photon with the energy *ħω*_2_ and wave vector **k**_**2**_. Hereby, the energy *ħω* = *ħω*_1_ − *ħω*_2_ and the momentum *ħ* **q** = *ħ* **k**_**1**_ − *ħ* **k**_**2**_ is transferred to the system by excitation of electrons from a core-shell state $|{\rm{i}}\rangle$ with energies *E*_*i*_ into an un-occupied state $|{\rm{f}}\rangle$ with energy *E*_*f*_. The scattered photons are analyzed via Bragg reflection often using a spherically bent analyzer crystal at a fixed energy and scattering angle 2*θ*. In order to measure the XRS spectrum across an absorption edge, the energy loss *ħω* is tuned in the vicinity of the electron binding energies by changing the incident energy when exploiting inverse geometry. The measured quantity in an XRS experiment is given by the double differential scattering cross section (DDSCS)^44^:

1 $$\frac{{{\rm{d}}}^{2}\sigma }{d{\rm{\Omega }}d{\omega }_{2}}={(\frac{{\rm{d}}\sigma }{d{\rm{\Omega }}})}_{{\rm{Th}}}S({\boldsymbol{q}},\omega \mathrm{)}.$$

Here, (*dσ* / *d*Ω)_Th_ is the Thomson cross section, which describes the coupling between the photons and electrons, *S*(**q**, *ω*) is the dynamic structure factor, which contains the information on all of electronic excitations in the system, and is defined as^44^:

2 $$S({\bf{q}},\omega )=\sum _{i,f}{p}_{i}| < {\rm{f}}|\sum _{j}{e}^{i{\bf{q}}\cdot {r}_{j}}|{\rm{i}} > {|}^{2}\cdot \delta ({E}_{f}-{E}_{i}-\hslash \omega ).$$

The delta function ensures the energy conservation and *p*_*i*_ is the probability for the initial state $|{\rm{i}}\rangle$. The cross section of such an event is very small compared to that of photoelectric absorption. Therefore, XRS measurements are typically conducted at 3rd generation synchrotron radiation sources. By changing the scattering angle 2*θ* and therefore the momentum transfer *q* different types of excitations can be probed. For very low *q*, dipole transitions dominate the signal and for high *q* the weight of non-dipole transitions increases^66–68^. Thorough overviews on this method can be found in^44,45,53,69–71^.

### Experimental details

The high pressure measurements of the M_2,3_-edges of siderite single crystal with argon as pressure medium were performed at beamline P01 of PETRAIII at DESY in Hamburg, Germany. All other *in situ* studies were carried out at the beamline ID20 of the European Synchrotron Radiation Facility in Grenoble, France using the large solid angle spectrometer^72^. In both cases, a multi-analyzer spectrometer employing the Si(880) analyzer reflection at 12.926 keV was used to measure the XRS signal by scanning the incident energy from 12.961 keV to 13.011 keV for the iron M_2,3_ and magnesium L_2,3_-edges and from 13.623 keV to 13.663 keV for the iron L_2,3_-edges with an overall energy resolution of 2.1 eV and 1.9 eV energy resolution at ID20 and P01, respectively. At ID20 the iron L_2,3_-edges were measured at low momentum transfer and iron M_2,3_ and magnesium L_2,3_-edges at high momentum transfer (corresponding to average scattering angles of 26.8° and 143.5° and momentum transfers of 3.2 ± 0.9 Å^−1^ and 12.5 ± 0.3 Å^−1^) with a beam size on the sample of 10 × 20 *μ*m^2^ using maxipix 2d detector with a pixel size of 55 *μ*m. The P01 spectra were measured at an average scattering angle of 155° and a momentum transfer of 12.8 ± 0.2 Å^−1^ with a beam size on the sample of 13 × 15 *μ*m^2^ using a LAMBDA 2d detector with a pixel size of 55 *μ*m. The spectra measured at ID20 were collected in 4–5 hours (M_2,3_-edges), in 12hours for the 2.4 GPa L_2,3_-edge and 7–8 hours for all other L_2,3_-edges. The M_2,3_-edges measured at P01 were collected in 3–4 hours. The FeCO_3_ and (Mg_0.74_ Fe_0.26_)CO_3_ single crystal synthesis is reported by Cerantola *et al*.^18^. For the Mg L_2,3_-edge measurements a natural MgCO_3_ powder was used. In order to expose the single crystals (size about 15*x*15*x*25 *μ*m^3^) to high pressure, we used Boehler-Almax plate diamond anvil cells (diamonds type Ia 300 *μ*m culet size, 80° opening angle, initial gasket hole diameter of about 150 *μ*m, and precompressed to 40 *μ*m), ESRF standard membrane cells (diamonds type Ia 250 *μ*m culet size, 80° degrees opening angle, initial gasket hole diameter of about 120 *μ*m, and precompressed to 35 *μ*m) and panoramic cells provided by the Extreme Conditions Support Infrastructure (ECSI) at DESY together with rhenium gaskets and helium/argon as pressure medium to guarantee quasi-hydrostatic conditions for the measurements. A ruby was loaded into the sample volume and we used the ruby fluorescence wavelength method^73^ to estimate the pressure before and after each experiment.

### Data extraction via XRS imaging

Owing to the small scattering cross section of XRS, the data extraction and background subtraction procedures are critical, standard procedures^53,74^ might not be applicable when DACs are used. However, recent developments in using area detectors in combination with spherically bent analyzer crystals open new possibilities for data treatment. By evaluating the measured intensity pixelwise along the beam path direction (shown in Fig. 1), one is able to select specific pixels on the detector, which collect signals from x-rays scattered by the sample, the sample environment, or both. By choosing a region of interest (ROI), the signal stemming solely from the sample can be extracted, which significantly increases the signal-to-noise ratio of the experiment. One has to keep in mind that at high pressures the sample volume decreases drastically and the pixel size may not be sufficient to strictly separate contributions from the sample and the environment. Hence, shifting the ROI by a few pixels enables extracting the background signal solely coming from the sample environment, which then can be used for the final background correction. Figure 1(b,c) shows an example of the thorough pixel-by-pixel analysis to extract the XRS signal at the iron M_2,3_-edge. In this case, the spectra C and D extracted from two detector pixels recording signal from the sample, while some neighboring pixels can be used to extract the background signal (curves A and B). This procedure of data analysis is a crucial necessity for handling this complex background. The weak scattering contribution from the diamond, partially masked by the rhenium gasket, is shown as curve E. Next, the background of the diamond (spectrum B) measured close to the sample is smoothed and subtracted from the signal that includes contributions from diamond and sample (C + D). The resulting spectrum of the iron M_2,3_-edge after background correction is shown in Fig. 1(d). This simplified background subtraction procedure has to be crosschecked by comparison of the spectra extracted at low pressure with a reference sample measured at ambient conditions (see e.g. Fig. 2(b)). In case that the shape of the diamond background strongly differs in the measured energy loss range from that of the contribution from valence and core electrons of the sample superimposing the corresponding M- or L-edge, latter have to be also considered within the background subtraction scheme^45^. The iron L_2,3_-edges have been extracted using the same approach. To enable comparison with literature spectra, for the L_2,3_-edge a double arctangent correction function was subtracted following ref.^41^.
